# Novel Small-molecule Antibacterials against Gram-positive Pathogens of *Staphylococcus* and *Enterococcus* Species

**DOI:** 10.3390/antibiotics8040210

**Published:** 2019-11-02

**Authors:** Marius Seethaler, Tobias Hertlein, Björn Wecklein, Alba Ymeraj, Knut Ohlsen, Michael Lalk, Andreas Hilgeroth

**Affiliations:** 1 Institute of Pharmacy, Martin-Luther-University Halle-Wittenberg, 06120 Halle, Germany; marius.seethaler@pharmazie.uni-halle.de; 2 Institute of Molecular Infection Biology, Julius Maximilians University Würzburg, 97080 Würzburg, Germany; tobias.hertlein@uni-wuerzburg.de (T.H.); bjoern.wecklein@stud-mail.uni-wuerzburg.de (B.W.); alba.ymeraj@stud-mail.uni-wuerzburg.de (A.Y.); knut.ohlsen@uni-wuerzburg.de (K.O.); 3 Institute of Biochemistry, Ernst-Moritz-Arndt-University Greifswald, 17489 Greifswald, Germany; lalk@uni-greifswald.de

**Keywords:** antibacterial activity, synthesis, substituent, structure-activity, inhibition

## Abstract

Defeat of the antibiotic resistance of pathogenic bacteria is one great challenge today and for the future. In the last century many classes of effective antibacterials have been developed, so that upcoming resistances could be met with novel drugs of various compound classes. Meanwhile, there is a certain lack of research of the pharmaceutical companies, and thus there are missing developments of novel antibiotics. Gram-positive bacteria are the most important cause of clinical infections. The number of novel antibacterials in clinical trials is strongly restricted. There is an urgent need to find novel antibacterials. We used synthetic chemistry to build completely novel hybrid molecules of substituted indoles and benzothiophene. In a simple one-pot reaction, two novel types of thienocarbazoles were yielded. Both indole substituted compound classes have been evaluated as completely novel antibacterials against the *Staphylococcus* and *Enterococcus* species. The evaluated partly promising activities depend on the indole substituent type. First lead compounds have been evaluated within in vivo studies. They confirmed the in vitro results for the new classes of small-molecule antibacterials.

## 1. Introduction

The increasing resistances of pathogenic bacteria are one great problem for worldwide health policy, because it means a threat of humanity [1]. Pathogenic bacteria can be found in human surroundings, and drinking water may be contaminated with bacteria in developing countries [2]. In the last century sufficient antibacterials were available to effectively treat infections with those various bacteria [1]. Beside the standard antibacterials like penicillin, reserve antibiotics have been discovered from natural sources and were resynthesized by industrial companies [3]. However, an abuse and overuse of those antibiotics is one of the reasons for the present crisis with resistant bacteria [1,3]. In the case of viral infections, antibiotics are often prescribed. In the case of existing sensitivities of bacteria towards standard antibiotics, novel reserve antibiotics have been used in clinics [3]. Therefore, bacteria with contact to those antibiotics like *Staphylococcus aureus* on human surfaces like skin developed early resistances [3,4,5]. A misuse of antibiotics is also present in the mast of poultry. Those antibiotics contaminate the environment by animal excretions [3,6]. Finally, industrial waste of antibiotic production is pumped into rivers in producing countries like India [1,7]. Moreover, bacteria develop resistance mechanisms that allow the spread of resistance genes via plasmids, and also between different species [8]. One other problem that contributes to the present antibiotic crisis is the fact that pharmaceutical companies recently announced that they would no longer invest in the development of novel antibiotics. The financial outcome of the development of life style drugs is much better than that of antibiotics. Antibiotics have to be cheap for a use in low-income or developing countries [1,2,3]. Innovative but vague approaches to find novel antibiotics have been an influencing of the bacterial gene expression to modify the bacterial metabolism [1]. Such a modified metabolism might lead to novel metabolic products which have to be identified and tested for their antibacterial properties. Methods to alter gene expression may be varied growth conditions or a microbial co-culturing of various bacteria or of fungi and bacteria [1,9,10,11]. Other sources for the identification of novel antibiotics have been plants, marine invertebrates and insects or amphibia [1,12,13,14,15]. Isolated antimicrobial peptides from such sources have long been favored. However, they still hamper with various problems like proteolytic instability, short in vivo half-lives, insufficient solubility and a poor bioavailability [1].

Alternative small-molecule antibacterials are rare. They own promising properties for an oral use, and are not expected to show problems reported for those antimicrobial peptides [1]. Presently, such small-molecule antibacterials that result from synthetic chemistry are unknown to emerge as a novel antibacterial compound class.

We gained access to two novel structurally-related classes of small-molecule antibacterials by the reaction of substituted indoles and thiophene dicarbaldehyde. Therefore, various composed hybrid molecules were yielded from those indoles and benzothiophene. Hybrid molecules of antimicrobial active antibiotics, or antifungal drugs have recently been reviewed [16,17]. They mostly consist either of two different antibiotics or antifungals that are connected by a linker like a simple carbon chain. So fluoroquinolone-oxazolidinone hybrids reached clinical trials and novel 1, 2, 3-triazole antifungal hybrids with 8-aminoquinoline or dioxolane have recently been reported [16,17,18,19].

Our novel compound classes were tested against prominent Gram-positive bacteria that are associated with severe hospital infections by *Staphylococcus* and *Enterococcus* strains. We identified first lead compounds with promising activities and confirmed the in vitro results first in the in vivo studies for selected derivatives.

## 2. Results and Discussion

### 2.1. Synthesis of the Thienocarbazoles

Benzo[*b*]thiophenes have been reported to show antimicrobial activities depending on their substituents [20]. The formation of all those benzo[*b*]thiophene compounds started from a 3- or 7- mostly halogen substituted benzo[*b*]thiophene scaffold which reacted with its 2-substituent to give 2-urea, -semicarbazide, -acyl or -heteroaryl substituted compounds [21,22,23,24]. Those added 2-substituents mostly owned substituted phenyl residues. However, a complex set of halogen, nitro or methoxy functions at the benzo[*b*]thiophene scaffold and the substituted phenyl residues was found necessary for such activities [20]. Indoles on the other hand have been reported to show some moderate antibacterial activities, depending on their heterocyclic substitution patterns [25]. Recent methods have been described to substitute benzo[*b*]thiophenes with aryl substituents, but methods to synthesize fused aryl benzo[*b*]thiophenes are rare [20]. Early methods were also successful to substitute carbazole with thiophene, but thienocarbazoles have not been reported so far [26]. So our approach to produce substituted benzo[*b*]thiophenes is completely different from earlier methods that concentrated on the benzo[*b*]thiophene derivatization.

**Scheme 1:**
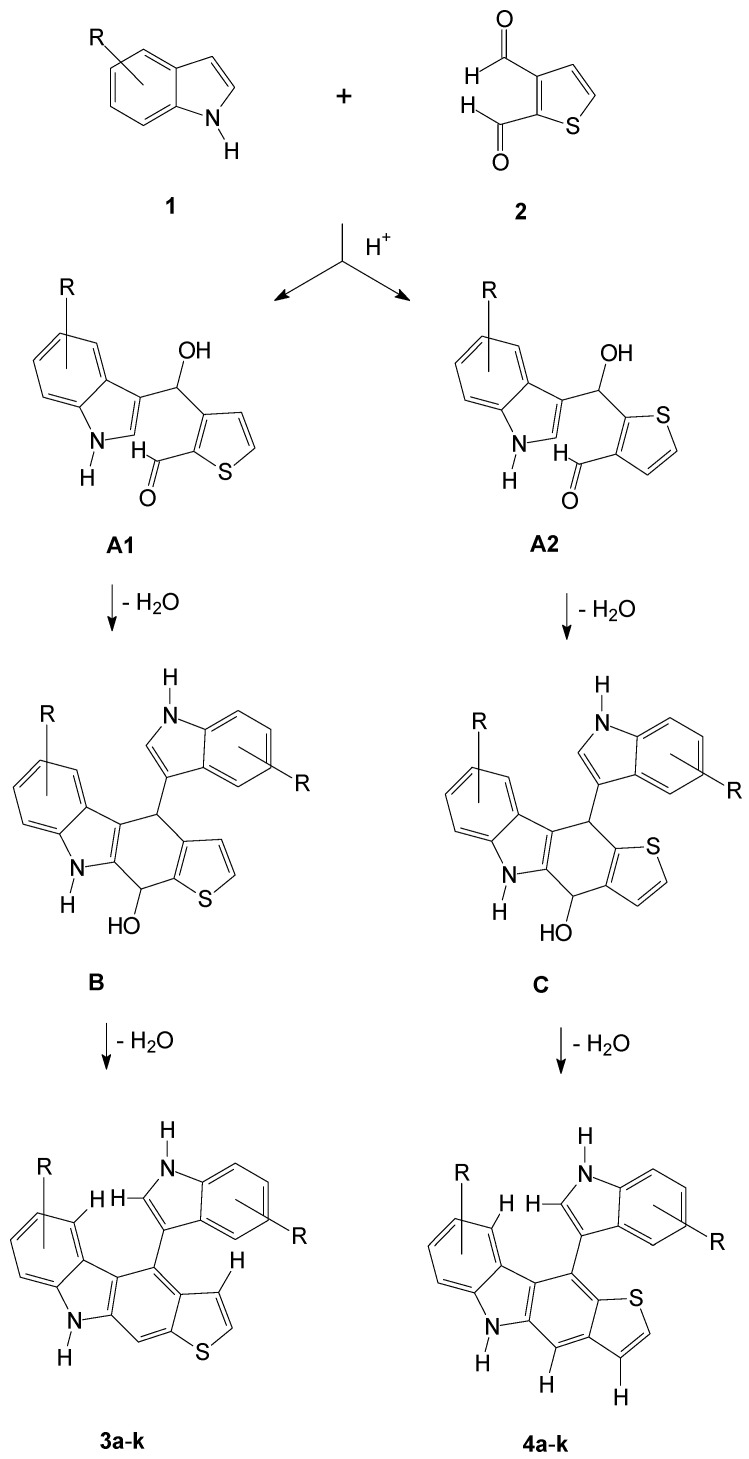
Formation of target compounds **3a**-**k** and **4a**-**k**.

We succeeded to synthesize two classes of thienocarbazoles **3** and **4** in a simple one-pot reaction of various substituted indoles and thiophene dicarbaldehyde. The suggested mechanism of the product formation under the used acidic conditions may be as follows (Scheme 1): One of the dialdehyde functions, either that at C-3 or that at C-2 of the thiophene **2**, is protonated to react with one electron-rich C-3 position of one indole **1**. Then the resulting either carbinol **A1** or **A2** is protonated again, and after water elimination the remaining carbenium is attacked by the C-3 of the second indole, whereas the remaining unreacted, but protonated, aldehyde function is attacked by the C-2 of the first reacted indole to give the two cyclized intermediates **B** and **C**. After a final water elimination, the two types of thienocarbazoles **3** and **4** result.

The structure of both compound classes **3** and **4** was verified by spectroscopic methods as follows: In the nuclear Overhauser effect (NOE) spectra of compounds **3**, we observed an NOE effect between the C2-hydrogen of the rotable attached indole function and both the neighbored C4-hydrogen of the incorporated indole function and the C4-hydrogen of the anelleated thiophene function. In the NOE spectra of compounds **4** we found only a NOE effect between the C2-hydrogen of the rotable attached indole and the neighbored C4-hydrogen of the incorporated indole. In compounds **4** we found an additional NOE effect between the aromatic C4-hydrogen of the molecular scaffold and the C3-hydrogen of the anellated thiophene function that was not observed in the spectra of compounds **3**. The discussed protons have been added to the respective compound structures in Scheme 1. We also tried to synthesize atropisomers by the use of 4-substituted indoles, so that a sterical hindrance of the indole rotability would result. However, due to that sterical hindrance in the approach of the reacting molecules, we failed to synthesize such compounds, although they would have been interesting concerning their antimicrobial activity.

### 2.2. In Vitro Antibacterial Activity of the Thienocarbazoles

As the reported benzo[*b*]thiophenes and the indoles showed activities towards *S. aureus*, we first investigated our compound classes as promising growth inhibitors of *S. aureus*. The determined minimal inhibitory concentration (MIC) values are shown in Table 1. Our selected indoles have been substituted in 5- and 6-positions to investigate a possible effect of the substituent position within the indole residue. Various antibiotics have been used for comparison.

The substituted indole compounds **3a** and **4a** showed moderate antibacterial activity towards *S. aureus* compared to the used antibiotics. A chloro-substitution in the 5-indole position mainly increased the activity for compounds **3b** and **4b**. Both reached the activity value of vancomycin. If moved to the 6-position of the indole we observed a slight decrease in activity for compounds **3c** and **4c** with MIC values of 8 µg/mL. A 5-bromo indole substitution in compound **3d** was less favorable than the 5-chloro substitution of derivative **3b**. If moved to the 6-indole position, the activity of compound **4e** was lower than that of the 6-chloro indole derivative **4c**. A 5-cyano indole function in compound **4f** was as favorable as the 5-chloro substitution with the MIC value of 4 µg/mL. In compound **3f** it was found less favorable with a decreased activity compared to compound **3b**. If moved to the 6-indole position in compounds **3g** and **4g**, the same activities were found. A 5-hydroxy function resulted in increased activities for derivative **3h**. The activity was better than that of vancomycin. The activity of derivative **4h** however was lower. The 6-hydroxy indole compounds **3i** and **4i** showed best activities for both compound classes with 2 µg/mL. If the 5-hydroxy functions were replaced with 5-benzyloxy functions in compounds **3j** and **4j**, the activities mainly decreased to 64 µg/mL. For the 6-benzyloxy indole compounds **3k** and **4k** the results were similar.

So it can be stated that the halogen substituted 5-position tends to be more favorable than the 6-position. Compounds **3** partly showed improved activities compared to compounds **4**.

Best results were conducted for the hydroxyl-substituted indole compounds that reached the activity of the used antibiotics. The best reported benzo[*b*]thiophenes with a 2-urea phenyl or -semicarbazide phenyl substitution showed mainly reduced activities with MIC values of 40 µg/mL [21]. Those with a 2-acyl heteroaryl substitution showed higher activities up to 3 µg/mL, depending on the substitution of the heteroaryl-attached phenyl-substituent [22,23]. Those best benzo[*b*]thiophenes with a 2-heteroaryl-phenyl substitution showed similar activities [24]. So our hydroxyl indole substituted derivatives mean an improvement with best activities of 2 µg/mL.

The promising results encouraged us to determine the compound activity also against the *Enterococcus* species. These *Enterococcus* species mainly contribute to clinical infections beside *Staphylococcus*. So immunocompromised patients get infected by *Enterococcus* species like *Enterococcus faecalis* or *faecium* [27,28]. With proceeding resistances of *Enterococcus* strains against vancomycin, there is an urgent need to find novel antibacterials against those critical *Enterococcus* species [29]. We determined the antibacterial activity of our compound classes against different types of vancomycin-resistant enterococci, namely *Enterococcus faecalis* (*vanB*) and *E. faecium* (*vanA*), and additionally against *E. casseliflavus* (*vanC*), which is often associated with vancomycin resistances.

The indole-unsubstituted compounds **3a** and **4a** showed a moderate activity against *E. faecalis*. However, that was much better than the vancomycin activity that was only found residual. For the 5-chloro indole substitution we found an improved activity for derivative **4b** with 8 µg/mL, whereas the activity of compound **3b** remained unchanged. The 6-chloro indole substitution was less favorable for both derivatives **3c** and **4c** with reduced activities. The 5-bromo substitution in compound **3d** resulted in a similar activity than the 5-chloro substitution. Also the 6-bromo substitution in compound **4e** was similarly effective than the 6-chloro substitution in compound **4c**. The activity of derivative **3e** was lower if compared to that of the 6-chloro compound **3c**. The 5-cyano substitution in compounds **3f** and **4f** resulted in similar activities than the 5-chloro substitutions. A movement of the cyano function to the 6-indole position in compounds **3g** and **4g** mainly reduced the activity, especially for compound **4g**. The 5- and 6-benzyoxy functions showed just residual activities in both compound classes for derivatives **3j** and **4j** and **3k** and **4k**, respectively, similar to the used vancomycin. Both 5- and 6-hydroxy functions almost resulted in moderate activities of compounds **3h**, **3i** and **4i**, whereas that of compound **4h** was found reduced. Similar to the determined *S. aureus* activities, we found reduced MIC values for the 6-halogen substituted compounds.

Next we evaluated the compound activities towards *Enterococcus faecium*. The unsubstituted compounds **3a** and **3b** were almost not active. However, the 5-chloro compounds **3b** and **4b** showed mainly increased activities with MIC values of 8 µg/mL. The 6-chloro indole substituted derivatives **3c** and **4c** showed different activities with that for **3c** being reduced and that for **4c** being unchanged.

The 5-bromo compound **3d** was as active as the 5-chloro compound **3b**, whereas both 6-bromo derivatives **3e** and **4e** showed a mainly reduced activity with MIC values of 64 µg/mL. The 5- and 6-cyano derivatives of both compound classes **3f** and **4f** and **3g** and **4g** were completely inactive. Similar results were evaluated for the 5- and 6-benzyloxy compounds **3j** and **4j** and **3k** and **4k**. The 5-hydroxy indole compounds **3h** and **4h** showed a moderate activity with 16 µg/mL. The 6-hydroxy compounds **3i** and **4i** showed an improved activity of 8 µg/mL. So the 5-halogen and 6-hydroxy-substituted compounds showed best *Enterococcus* activities compared to the used vancomycin that was found not active against the *Enterococcus faecium* strain.

Finally, we determined the compound activity towards *Enterococcus casseliflavus*. Similar to the results against *Enterococcus faecium*, we found no compound activity for the unsubstituted compounds **3a** and **4a** and for both 5- and 6-substituted cyano and the 6-benzyloxy derivatives **3f**, **4f**, **3g**, **4g**, **3k** and **4k** with those of the 5-benzyloxy compounds **3j** and **4j** being residual. Concerning the 5-halogen substitutions, the best results were found for the 5-chloro derivatives **3b** and **4b** with MIC values of 8 µg/mL. The activities of the other halogen-substituted compounds of compound class **3** were moderate, with each at 16 µg/mL, whereas those of the compound class **4** were reduced with 32 µg/mL for compound **4c** and just 64 µg/mL for compound **4e**. Also the hydroxyl indole substitutions were not favorable, with 32 µg/mL for **3i** and **4i** and residual with 64 µg/mL for **3h** and **4h**. Best activities towards *Enterococcus casseliflavus* were found for the halogen-substituted derivatives of compound class **3** that all exceed that of the vancomycin control.

### 2.3. In Vivo Antibacterial Activity of Thieno[b]carbazoles

**Figure 1:**
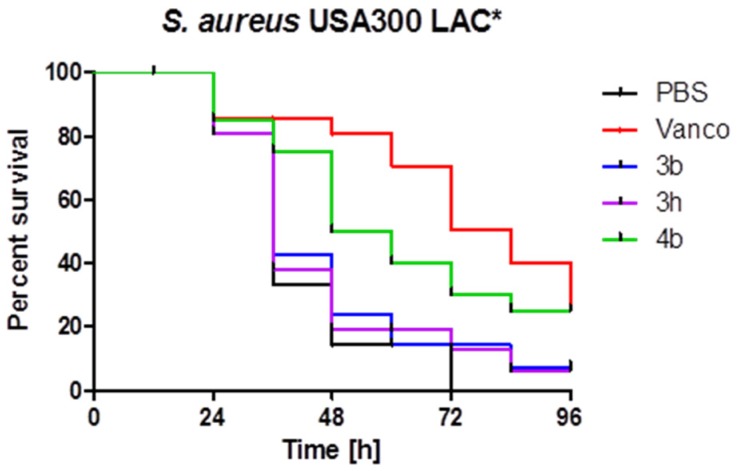
Survival of *Galleria mellonella* when infected with *S. aureus* strain USA300 LAC* and treated with **3b**, **3h**, **4b** or controls (phosphate-buffered saline (PBS), vancomycin), respectively (Kaplan-Meier plot).

Furthermore, the drug efficacy of compounds **3b, 3h** and **4b**, which showed high activities in vitro against *S. aureus* and vancomycin-resistant enterococci, was studied in a *Galleria mellonella* larvae in vivo model. *Galleria mellonella* larvae were infected with an *S. aureus* strain USA300 LAC* or vancomycin-resistant *E. faecalis* strain ATCC51299. As our control, the antibiotic vancomycin was used. Larvae infected with the MRSA strain USA300 LAC* showed an overall survival rate of 0% after 72% (Figure 1). However, when treated with **4b**, a significantly better survival of 30% was observed, as compared to the infected control group after 72 h (*p* ≤ 0.05; Figure 1). Compound **3b** and **3h** treatment insignificantly increased the survival to 14% and 13%, respectively after 72 h. Vancomycin treatment resulted in 50% survival after 72 h and 25% survival after 96 h. At the latest time point 96 h, **4b** showed the same survival rate as the control antibiotic vancomycin (both 25%). In contrast, in vivo activity against *E. faecalis* was not significant for all tested compounds, including vancomycin (data not shown). The median survival time was 42 h in the PBS negative control group, 36 h for vancomycin **3b** and **3h**, respectively, and 48 h for compound **4b**. Survival of larvae after 96 h was 0% in the phosphate-buffered saline (PBS), **3h** and **4b** group, respectively, 5% in the vancomycin group, and 7% in the **3b** group.

## 3. Materials and Methods

### 3.1. Chemical Reagents and Instruments

Commercial reagents were used without further purification. The ^1^H-NMR spectra (500 MHz) were measured using tetramethylsilane as the internal standard. Thin layer chromatography (TLC) was performed on E. Merck 5554 silica gel plates. The high-resolution mass spectra were recorded on a Finnigan LCQ Classic mass spectrometer.

### 3.2. Procedure for the Synthesis of Compound Classes 3 and 4

Each 2 mmol of indole and 1 mmol of the thiophene dicarbaldehyde were dissolved in 15 mL of acetic acid. The reaction mixture was stirred under heating at 100 °C and the reaction proceeding was followed by TLC. The reaction was stopped when no more of the indole starting product was detectable. The solution was neutralized with a 2.5 M solution of sodium hydroxide. The product mixture was filtered off from the solution, washed with portions of water, and then resolved in ethyl acetate. The organic layer was dried over sodium sulfate, filtered again and evaporated in vacuum. The remaining oil was purified by column chromatography, using silica gel and an eluent mixture of ethyl acetate and cyclohexane in a relation of 1:1 or 1:4, depending on the indole substitution. The product fractions containing compounds **3** and **4**, respectively, were collected and unified. The unified compound fractions were evaporated to give the solid compounds for analysis. Spectroscopical data have been summarized in the Supplementary Materials.

### 3.3. In Vitro Antibacterial Activity

The compounds and the used antibiotics were dissolved in 12.5% dimethyl sulfoxide (DMSO) at a concentration of 512 µg/mL. Further dilutions of the compounds and standard drugs in the test medium were prepared at the required quantities of 256, 128, 64, 32, 16, 8, 4, 2, 1, 0.5 and 0.25 µg/mL concentrations with Mueller-Hinton broth. The minimum inhibitory concentrations (MIC) were determined using the 2-fold serial dilution technique. All the compounds were tested for their in vitro growth inhibitory activity against *S. aureus* USA300 LAC*, the *Enterococcus faecium* strain UL602570, *Enterococcus faecalis* strain ATCC51299, and *Enterococcus casseliflavus* Z700327 strain. 

All strains were cultivated from the strain collection of the Institute of Molecular Infection Biology, University of Würzburg.

The cultures were obtained from Mueller-Hinton broth (Difco) for all the bacterial strains after 24 h of incubation at 37 ± 1 °C. Testing was carried out in Mueller-Hinton broth at pH 7.4 and the 2-fold serial dilution technique was applied using microtiter plates. Each test well was inoculated with 100 µL of the respective compound and 100 µL bacterial suspension. The final inoculum size was 5 × 10^5^ CFU/mL for the antibacterial assay. A set of wells containing only inoculated broth was used as control. After incubation for 24 h at 37 ± 1 °C, the last well with no growth of microorganism was recorded to represent the MIC (expressed in µg/mL).

### 3.4. In Vivo Antibacterial Assay

To evaluate the antibacterial activity of the compounds in vivo, *Galleria mellonella* larvae (Feeders & more GmbH, Au in der Hallertau, Germany) were infected with *S. aureus* USA300 LAC* and *E. faecalis* ATCC51299 and treated with different compounds. The bacteria were prepared from a fresh culture which had reached an OD600 of 0.6. The culture was harvested (3200× *g*, 10 min, 4 °C) and washed once with 20 mL 1× PBS (3200× *g*, 10 min, 4 °C). The bacteria were then diluted to the desired infection dose of 2.5 × 10^7^ CFU/mL for *S. aureus* LAC* and 5 × 10^7^ CFU/mL for *E. faecalis* ATCC51299. 20 μL of the prepared bacteria were injected into the hemocoel through the final pair of prolegs. 1 h after the infection the larvae were treated with 20 μL of compounds diluted in 1× PBS with a final concentration of 128 μg/mL and were then incubated at 37 °C. The larvae were monitored for 4 days and checked every 12 h; survival curves were generated using GraphPad Prism. The experiment was performed with 20 larvae for each compound as well as the controls. The larvae were kept in Petri dishes, as groups of 10. 1× PBS was used as the negative control while vancomycin was used as the positive control. Larvae were recorded as dead when they were colored black, or not moving after stimulation with a pair of tweezers.

## 4. Conclusions

Over- and misuse of antibiotics, as well as contamination of the environment with antibiotics and antibiotic waste increase antibacterial resistances. Industrial efforts to develop novel antibiotics have been strictly shortened, so that almost just variations of established antibiotics are available. In case of the increasing resistances against the Gram-positive bacteria *Staphylococcus aureus* and *Enterococcus* species, those cost-intensive variations of known antibiotics partly do not justify their use because of a limited outcome concerning expected resistances [4]. Presently, just two novel antibacterials undergo clinical trials [4]. So there is a strong need for novel antibacterials with novel structures. We developed novel small-molecule antibacterials of two compound classes by a simple one-pot reaction of two components. That simple compound access means an effective low-cost production. In both different thieno[*b*]carbazole classes, promising candidates have been identified. They showed best activities towards *Staphylococcus* in case of a 5-chloro, a 6-cyano and both a 5- and 6-hydroxy indole substitution Most promising compounds in case of the various *Enterococcus* species have been those with a 5-chloro or -bromo as well as the 6-hydroxy indole substitution. Their activities farly exceed those of the used standard vancomycin. For selected compounds the in vitro results could be confirmed in an in vivo galleria infection model. As they are practically new small-molecule antibacterials, we expect our contribution in the field to be hopeful for further studies. These studies will partly concentrate on the identification of the bacterial target which is addressed by our novel compounds. One suspected target structure for consideration is the bacterial pyruvate kinase that is addressed by structurally-related bis-indolyl derivatives similar to the marine bis-indolyl alkaloid dihydrohamacanthin [30].

## Figures and Tables

**Table 1:** Minimal inhibitory concentration (MIC) values determined for target compounds **3a**–**k** and **4a**–**k** in *Staphylococcus aureus* USA300 LAC* and *Enterococcus* species.

		MIC [µg/mL] ^a^			
**Cpd.**	R	*Staphylococcus aureus*	*E. faecium*	*E. faecalis*	*E. casseliflavus*
**3a**	H	8	128	16	>128
**3b**	5-Cl	4	8	16	8
**3c**	6-Cl	8	32	32	16
**3d**	5-Br	8	8	16	16
**3e**	6-Br	-	64	64	16
**3f**	5-CN	8	>128	32	128
**3g**	6-CN	4	>128	64	>128
**3h**	5-OH	2	16	16	64
**3i**	6-OH	2	8	16	32
**3j**	5-OBn	64	>128	128	64
**3k**	6-OBn	64	>128	128	128
**4a**	H	8	>128	16	>128
**4b**	5-Cl	4	8	8	8
**4c**	6-Cl	8	8	32	32
**4e**	6-Br	16	64	32	64
**4f**	5-CN	4	>128	32	>128
**4g**	6-CN	4	>128	128	>128
**4h**	5-OH	8	16	32	64
**4i**	6-OH	2	8	16	32
**4j**	5-OBn	64	>128	128	64
**4k**	6-OBn	32	>128	128	128
Oxacillin		1	-	-	-
Ciprofloxacin		-	-	4	-
Ampicillin		-	>128	2	-
Vancomycin		4	>128	128	32

^a^ Mean of three determinations.

## Supplementary Materials

The following are available online at https://www.mdpi.com/2079-6382/8/4/210/s1, Data S1: Spectroscopical compound data.
