# Supplementary material for: Novel Small-molecule Antibacterials against Gram-positive Pathogens of Staphylococcus and Enterococcus Species
Source: Antibiotics (Basel). 2019 Nov 2;8(4):210. doi: 10.3390/antibiotics8040210 (PMC6963286; doi:10.3390/antibiotics8040210)
Supplement: Supplementary file 1 [file antibiotics-08-00210-s001.pdf]

Supplementary Materials

Novel small-molecule antibacterials against Gram-positive pathogens of *Staphylococcus* and *Enterococcus* species

Marius Seethaler [1], Tobias Hertlein [2], Knut Ohlsen [2], Björn Wecklein [2], Alba Ymeraj [2], Michael Lalk [3] and Andreas Hilgeroth [1,*]

[1] Institute of Pharmacy, Martin-Luther-University Halle-Wittenberg, 06120 Halle, Germany; andreas.hilgeroth@pharmazie.uni-halle.de
[2] Institute of Molecular Infection Biology, Julius Maximilians University Würzburg, 97080 Würzburg, Germany
[3] Institute of Biochemistry, Ernst-Moritz-Arndt-University Greifswald, 17489 Greifswald, Germany
* Correspondence: andreas.hilgeroth@pharmazie.uni-halle.de; Tel.: +49-345-55-25168

4-(1H-Indol-3-yl)-9H-thieno[2,3-b]carbazole (**3a**). Yield 35%, white powder; mp 242-246 °C; ^{1}H NMR (DMSO-d_6) δ = 11.50 (s, br, 1H, N′-H), 11.26 (s, 1H, NH), 8.00 (d, *J* = 0.8 Hz, 1H, 10-H), 7.64 (d, *J* = 2.4 Hz, 1H, 2′-H), 7.57 (d, *J* = 8.2 Hz, 1H, 7′-H), 7.42 (d, *J* = 7.1 Hz, 1H, 8-H), 7.40 (d, *J* = 5.2 Hz, 1H, 2-H), 7.24 (dd, *J* = 8.2, 7.1 Hz, 1H, 7-H), 7.16 (dd, *J* = 8.2, 6.7 Hz, 1H, 6′-H), 7.11 (dd, *J* = 5.5, 0.8 Hz, 1H, 3-H), 7.02 (d, *J* = 7.2 Hz, 1H, 5-H), 6.97 (d, *J* = 7.9 Hz, 1H, 4′-H), 6.90 (dd, *J* = 7.9, 6.7 Hz, 1H, 5′-H), 6.72 (dd, *J* = 8.2, 7.2 Hz, 1H, 6-H); *m/z* (ESI) 337.49 (M-H$^+$).

6-Chloro-4-(5-chloro-1H-indol-3-yl)-9H-thieno[2,3-b]carbazole (**3b**). Yield 74%, white powder; mp 230-233 °C; ^{1}H NMR (DMSO-d_6) δ = 11.41 (s, br, 1H, N′-H), 11.00 (s, 1H, NH), 8.54 (d, *J* = 0.8 Hz, 1H, 10-H), 8.00 (d, *J* = 2.4 Hz, 1H, 2′-H), 8.13 (d, *J* = 8.6 Hz, 1H, 7′-H), 7.94 (d, *J* = 8.5 Hz, 1H, 8-H), 7.84 (d, *J* = 5.6 Hz, 1H, 2-H), 7.74 (dd, *J* = 8.5, 2.1 Hz, 1H, 7-H), 7.67 (dd, *J* = 8.6, 2.1 Hz, 1H, 6′-H), 7.62 (dd, *J* = 5.6, 0.8 Hz, 1H, 3-H), 7.59 (d, *J* = 2.1 Hz, 1H, 5-H), 7.53 (d, *J* = 2.1 Hz, 1H, 4′-H); *m/z* (ESI) 405.31 (M-H$^+$).

7-Chloro-4-(6-chloro-1H-indol-3-yl)-9H-thieno[2,3-b]carbazole (**3c**). Yield 49%, white powder; mp 250-253 °C; ^{1}H NMR (DMSO-d_6) δ = 11.68 (s, br, 1H, N′-H), 11.45 (s, 1H, NH), 8.06 (d, *J* = 0.8 Hz, 1H, 10-H), 7.72 (d, *J* = 2.5 Hz, 1H, 2′-H), 7.61 (d, *J* = 1.2 Hz, 1H, 7′-H), 7.46 (d, *J* = 2.2 Hz, 1H, 8-H), 7.45 (d, *J* = 5.4 Hz, 1H, 2-H), 7.10 (dd, *J* = 5.4, 0.8 Hz, 1H, 3-H), 6.92 (m, 2H, 4′-, 5′-H), 6.91 (d, *J* = 8.4 Hz, 1H, 5-H), 6.81 (dd, *J* = 8.4, 2.2 Hz, 1H, 6-H); *m/z* (ESI) 405.52 (M-H$^+$).

6-Bromo-4-(5-bromo-1H-indol-3-yl)-9H-thieno[2,3-b]carbazole (**3d**). Yield 74%, white powder; mp 212-215 °C; ^{1}H NMR (DMSO-d_6) δ = 11.81 (d, *J* = 2.6 Hz, 1H, N′-H), 11.51 (s, 1H, NH), 8.08 (d, *J* = 0.8 Hz, 1H, 10-H), 7.77 (d, *J* = 2.6 Hz, 1H, 2′-H), 7.58 (d, *J* = 8.5 Hz, 1H, 7′-H), 7.48 (d, *J* = 5.6 Hz, 1H, 2-H), 7.43-7.39 (m, 2H, 7-, 8-H), 7.31 (dd, *J* = 8.5, 1.9 Hz, 1H, 6′-H), 7.09-7.06 (m, 2H, 3-, 5-H), 7.05 (d, *J* = 1.9 Hz, 1H, 4′-H); *m/z* (ESI) 495.39 (M-H$^+$).

7-Bromo-4-(6-bromo-1H-indol-3-yl)-9H-thieno[2,3-b]carbazole (**3e**). Yield 68%, yellow powder; mp 239-242 °C; ^{1}H NMR (DMSO-d_6) δ = 11.68 (d, *J* = 2.4 Hz, 1H, N′-H), 11.45 (s, 1H, NH), 8.07 (d, *J* = 0.8 Hz, 1H, 10-H), 7.75 (d, *J* = 1.7 Hz, 1H, 7′-H), 7.71 (d, *J* = 2.4 Hz, 1H, 2′-H), 7.60 (d, *J* = 1.8 Hz, 1H, 8-H), 7.46 (d, *J* = 5.6 Hz, 1H, 2-H), 7.10 (dd, *J* = 5.6, 0.8 Hz, 1H, 3-H), 7.04 (dd, *J* = 8.5, 1.8 Hz, 1H, 6-H), 6.93 (dd, *J* = 8.5, 1.7 Hz, 1H, 5′-H), 6.88 (d, *J* = 8.5 Hz, 1H, 5-H), 6.85 (d, *J* = 7.9 Hz, 1H, 4′-H); *m/z* (ESI) 495.38 (M-H$^+$).

4-(5-Cyano-1H-indol-3-yl)-9H-thieno[2,3-b]carbazole-6-carbonitrile (**3f**). Yield 64%, white powder; mp 206-209 °C; ^{1}H NMR (DMSO-d_6) δ = 12.22 (d, *J* = 2.5 Hz, 1H, N′-H), 12.00 (s, 1H, NH), 8.20 (d, *J* = 0.8 Hz, 1H, 10-H), 8.00 (d, *J* = 2.5 Hz, 1H, 2′-H), 7.80 (dd, *J* = 8.5, 0.7 Hz, 1H, 7′-H), 7.67 (d, *J* = 8.4 Hz, 1H, 7-H), 7.61 (dd, *J* = 8.4, 0.7 Hz, 1H, 8-H), 7.57 (dd, *J* = 8.5, 1.5 Hz, 1H, 6′-H), 7.56 (d, *J* = 5.6 Hz, 1H, 2-H), 7.41 (dd, *J* = 1.4, 0.7 Hz, 1H, 5-H), 7.17 (dd, *J* = 1.5 Hz, 0.7 Hz, 1H, 4′-H), 7.11 (dd, *J* = 5.6, 0.8 Hz, 1H, 3-H); *m/z* (ESI) 387.54 (M-H$^+$).

4-(6-Cyano-1H-indol-3-yl)-9H-thieno[2,3-b]carbazole-7-carbonitrile (**3g**). Yield 48%, yellow powder; mp 260-263 °C; ^{1}H NMR (DMSO-d_6) δ = 12.19 (d, *J* = 2.6 Hz, 1H, N′-H), 11.81 (s, 1H, NH), 8.18 (d, *J* = 0.8 Hz, 1H, 10-H), 8.10 (d, *J* = 1.5 Hz, 1H, 7′-H), 8.04 (d, *J* = 2.6 Hz, 1H, 2′-H), 7.91 (d, *J* = 1.5 Hz, 1H, 8-H), 7.52 (d, *J* = 5.6 Hz, 1H, 2-H), 7.25 (dd, *J* = 8.3, 1.5 Hz, 1H, 6-H), 7.17 (dd, *J* = 8.2, 1.5 Hz, 1H, 5′-H), 7.09 (dd, *J* = 5.6, 0.8 Hz, 1H, 3-H), 7.08 (d, *J* = 8.2 Hz, 1H, 4′-H), 6.99 (d, *J* = 8.3 Hz, 1H, 5-H); *m/z* (ESI) 387.53 (M-H$^+$).

4-(5-Hydroxy-1H-indol-3-yl)-9H-thieno[2,3-b]carbazol-6-ol (**3h**). Yield 43%, yellow powder; mp > 226 °C; ^{1}H NMR (DMSO-d_6) δ = 11.17 (d, *J* = 2.5 Hz, 1H, N′-H), 10.87 (s, 1H, NH), 8.55 (s, 1H, OH), 8,48 (s, 1H, OH), 7.89 (d, *J* = 0.8 Hz, 1H, 10-H), 7.47 (d, *J* = 2.5 Hz, 1H, 2′-H), 7.35 (d, *J* = 8.5 Hz, 1H, 7′-H), 7.34 (d, *J* = 5.6 Hz, 1H, 2-H), 7.21 (d, *J* = 8.5 Hz, 1H, 8-H), 7.02 (dd, *J* = 5.6, 0.8 Hz, 1H, 3-H), 6.78 (dd, *J* = 8.5, 2.4 Hz, 1H, 7-H), 6.67 (dd, *J* = 8.5, 2.4 Hz, 1H, 6′-H), 6.61 (d, *J* = 2.4 Hz, 1H, 5-H), 6.33(d, *J* = 2.4 Hz, 1H, 4′-H); *m/z* (ESI) 369.47 (M-H$^+$).

*4-(6-Hydroxy-1H-indol-3-yl)-9H-thieno[2,3-b]carbazol-7-ol (**3i**).* Yield 54%, white powder; mp 188-191 °C; ^{1}H NMR (DMSO-d_6) δ = 11.02 (d, *J* = 2.3 Hz, 1H, N′-H), 11.94 (s, 1H, NH), 9.30 (s, 1H, OH), 8.97 (s, 1H, OH), 7.85 (d, *J* = 0.8 Hz, 1H, 10-H), 7.34 (d, *J* = 2.3 Hz, 1H, 2′-H), 7.34 (d, *J* = 5.5 Hz, 1H, 2-H), 7.09 (dd, *J* = 5.5, 0.8 Hz, 1H, 3-H), 6.89 (d, *J* = 2.2 Hz, 1H, 7′-H), 6.86 (d, *J* = 8.6 Hz, 1H, 5-H), 6.75 (d, *J* = 2.0 Hz, 1H, 8-H), 6.73 (d, *J* = 8.5 Hz, 1H, 4′-H), 6.43 (dd, *J* = 8.5, 2.2 Hz, 1H, 5′-H), 6.22 (dd, *J* = 8.6, 2.0 Hz, 1H, 6-H); *m*/*z* (ESI) 369.49 (M-H$^+$).

*6-Benzyloxy-4-(5-benzyloxy-1H-indol-3-yl)-9H-thieno[2,3-b]carbazole (**3j**).* Yield 77%, white powder; mp 199-202 °C; ^{1}H NMR (DMSO-d_6) δ = 11.44 (d, *J* = 2.6 Hz, 1H, N′-H), 11.05 (s, 1H, NH), 7.95 (d, *J* = 0.8 Hz, 1H, 10-H), 7.60 (d, *J* = 2.6 Hz, 1H, 2′-H), 7.54 (dd, *J* = 5.7, 0.8 Hz, 1H, 7′-H), 7.38 (d, *J* = 5.7 Hz, 1H, 2-H), 7.31 (d, *J* = 8.7 Hz, 1H, 8-H), 7.28-7.17 (m, 8H, phenylic H), 7.15 (dd, *J* = 5.7, 0.8 Hz, 1H, 3-H), 7.10 (dd, *J* = 6.4, 1.9 Hz, 2H, *o*-phenylic H), 6.97 (dd, *J* = 8.7, 2.6 Hz, 1H, 7-H), 6.95 (dd, *J* = 8.8, 2.4 Hz, 1H, 6′-H), 6.69 (d, *J* = 2.6 Hz, 1H, 5-H), 6.57 (d, *J* = 2.4 Hz, 1H, 4′-H), 4.78 (q, *J* = 12.0 Hz, 2H, OCH_2), 4.55 (q, *J* = 12.0 Hz, 2H, OCH_2),; *m*/*z* (ESI) 549.21 (M-H$^+$).

*7-Benzyloxy-4-(6-benzyloxy-1H-indol-3-yl)-9H-thieno[2,3-b]carbazole (**3k**).* Yield 47%, white powder; mp 217-220 °C; ^{1}H NMR (DMSO-d_6) δ = 11.27 (d, *J* = 2.4 Hz, 1H, N′-H), 11.14 (s, 1H, NH), 7.92 (d, *J* = 0.8 Hz, 1H, 10-H), 7.51-7.47 (m, 2H, *o*-phenylic H), 7.47 (d, *J* = 2.4 Hz, 1H, 2′-H), 7.46-7.42 (m, 2H, *o*-phenylic H), 7.37 (d, *J* = 5.6 Hz, 1H, 2-H), 7.42-7.27 (m, 6H, *m*-, *p*-phenylic H), 7.12 (d, *J* = 2.3 Hz, 1H, 7′-H), 7.10 (dd, *J* = 5.6, 0.8 Hz, 1H, 3-H), 6.98(d, *J* = 2.3 Hz, 1H, 8-H), 6.92 (d, *J* = 8.7 Hz, 1H, 5-H), 6.84 (d, *J* = 8.6 Hz, 1H, 4′-H), 6.65 (dd, *J* = 8.6, 2.3 Hz, 1H, 5′-H), 6.47 (dd, *J* = 8.7, 2.3 Hz, 1H, 6-H), 5.15 (s, 2H, OCH_2), 5.11 (s, 2H, OCH_2); *m*/*z* (ESI) 549.26 (M-H$^+$).

*10-(1H-Indol-3-yl)-5H-thieno[3,2-b]carbazole (**4a**).* Yield 31%, pale yellow powder; mp 286-289 °C; ^{1}H NMR (DMSO-d_6) δ = 11.54 (s, br, 1H, N′-H), 11.26 (s, 1H, NH), 7.89 (s, 1H, 4-H), 7.73 (d, *J* = 2.5 Hz, 1H, 2′-H), 7.65 (d, *J* = 5.5 Hz, 1H, 2-H), 7.58 (dd, *J* = 8.2, 1.0 Hz, 1H, 7′-H), 7.53 (d, *J* = 5.5, 1H, 3-H), 7.42 (d, *J* = 8.2 Hz, 1H, 6-H), 7.25 (dd, *J* = 8.2, 7.1 Hz, 1H, 6′-H), 7.17 (dd, *J* = 8.2, 6.9 Hz, 1H, 7-H), 6.97 (d, *J* = 7.9 Hz, 2H, 4′-, 9-H), 6.90 (ddd, *J* = 7.9, 6.8, 1.0 Hz, 1H, 5′-H), 6.72 (dd, *J* = 7.9, 6.9 Hz, 1H, 8-H); *m*/*z* (ESI) 337.48 (M-H$^+$).

*8-Chloro-10-(5-chloro-1H-indol-3-yl)-9H-thieno[3,2-b]carbazole (**4b**).* Yield 14%, white powder; mp 266-269 °C; ^{1}H NMR (DMSO-d_6) δ = 11.44 (s, br, 1H, N′-H), 11.00 (s, 1H, NH), 8.46 (s, 1H, 4-H), 8.30 (d, *J* = 2.5 Hz, 1H, 2′-H), 8.14 (d, *J* = 8.5 Hz, 1H, 7′-H), 8.08 (d, *J* = 5.5 Hz, 1H, 2-H), 7.98 (d, *J* = 5.5 Hz, 1H, 3-H), 7.94 (d, *J* = 8.5 Hz, 1H, 6-H), 7.74 (dd, *J* = 8.6, 2.1 Hz, 1H, 7-H), 7.68 (dd, *J* = 8.5, 2.1 Hz, 1H, 6′-H), 7.58-7.50 (m, 2H, 9-, 4′-H); *m*/*z* (ESI) 405.56 (M-H$^+$).

*7-Chloro-10-(6-chloro-1H-indol-3-yl)-9H-thieno[3,2-b]carbazole (**4c**).* Yield 37%, white powder; mp 179-182 °C; ^{1}H NMR (DMSO-d_6) δ = 11.72 (s, br, 1H, N′-H), 11.46 (s, 1H, NH), 7.94 (s, 1H, 4-H), 7.81 (d, *J* = 2.3 Hz, 1H, 2′-H), 7.69 (d, *J* = 5.5 Hz, 1H, 2-H), 7.62 (d, *J* = 1.2 Hz, 1H, 7′-H), 7.55 (d, *J* = 5.5 Hz, 1H, 3-H), 7.46 (d, *J* = 1.9 Hz, 1H, 6-H), 6.96-6.92 (m, 2H, 4′-, 5′-H), 6.89 (d, *J* = 8.5 Hz, 1H, 9-H), 6.81 (dd, *J* = 8.5, 1.9 Hz, 1H, 8-H); *m*/*z* (ESI) 405.59 (M-H$^+$).

*7-Bromo-10-(6-bromo-1H-indol-3-yl)-9H-thieno[3,2-b]carbazole (**4e**).* Yield 20%, yellow powder; mp 164-167 °C; ^{1}H NMR (DMSO-d_6) δ = 11.72 (d, *J* = 2.5 Hz, 1H, N′-H), 11.45 (s, 1H, NH), 7.94 (s, 1H, 4-H), 7.80 (d, *J* = 2.5 Hz, 1H, 2′-H), 7.62 (d, *J* = 1.8 Hz, 1H, 7′-H), 7.70 (d, *J* = 5.5 Hz, 1H, 2-H), 7.60 (d, *J* = 1.8 Hz, 1H, 6-H), 7.55 (d, *J* = 5.5 Hz, 1H, 3-H), 7.04 (dd, *J* = 8.5, 1.8 Hz, 1H, 5′-H), 6.93 (dd, *J* = 8.4, 1.8 Hz, 1H, 8-H), 6.88 (d, *J* = 8.5 Hz, 1H, 4′-H), 6.83 (d, *J* = 8.4 Hz, 1H, 9-H); *m*/*z* (ESI) 495.36 (M-H$^+$).

*10-(5-Cyano-1H-indol-3-yl)-9H-thieno[3,2-b]carbazole-8-carbonitrile (**4f**).* Yield 11%, white powder; mp 314-317 °C; ^{1}H NMR (DMSO-d_6) δ = 11.40 (s, br, 1H, N′-H), 11.03 (s, 1H, NH), 8.12 (s, 1H, 4-H), 8.06 (d, *J* = 2.6 Hz, 1H, 2′-H), 7.90 (dd, *J* = 8.5, 0.8 Hz, 1H, 7′-H), 7.71 (d, *J* = 5.6 Hz, 1H, 2-H), 7.68 (dd, *J* = 8.5, 0.8

Hz, 1H, 6-H), 7.64 (dd, *J* = 8.5, 1.6 Hz, 1H, 7-H), 7.60 (d, *J* = 5.6 Hz, 1H, 3-H), 7.59 (dd, *J* = 8.5, 1.6 Hz, 1H, 6′-H), 7.56 (dd, *J* = 1.6, 0.8 Hz, 1H, 4′-H), 7.39 (dd, *J* = 1.6, 0.8 Hz, 1H, 9-H); *m/z* (ESI) 387.48 ($M-H^+$).

*10-(6-Cyano-1H-indol-3-yl)-9H-thieno[3,2-b]carbazole-7-carbonitrile (**4g**).* Yield 37%, yellow powder; mp 212-215 °C; ^1H NMR (DMSO-d_6) δ = 12.23 (d, J = 2.7 Hz, 1H, N′-H), 11.81 (s, 1H, NH), 8.13 (d, J = 2.7 Hz, 1H, 2′-H), 8.11 (d, J = 1.8 Hz, 1H, 7′-H), 8.04 (s, 1H, 4-H),), 7.91 (d, J = 1.4 Hz, 1H, 6-H), 7.78 (d, J = 5.5 Hz, 1H, 2-H), 7.61 (d, J = 5.5 Hz, 1H, 3-H), 7.26 (dd, J = 8.3, 1.8 Hz, 1H, 5′-H), 7.18 (dd, J = 8.2, 1.4 Hz, 1H, 8-H), 7.09 (d, J = 8.3 Hz, 1H, 4′-H), 6.98 (d, J = 8.2 Hz, 1H, 9-H); m/z (ESI) 387.52 ($M-H^+$).

*10-(5-Hydroxy-1H-indol-3-yl)-9H-thieno[3,2-b]carbazol-8-ol (**4h**).* Yield 24%, yellow powder; mp > 228 °C; ^1H NMR (DMSO-d_6) δ = 11.20 (d, *J* = 2.6 Hz, 1H, N′-H), 10.86 (s, 1H, NH), 8.53 (s, 1H, OH), 8.47 (s, 1H, OH), 7.79 (s, 1H, 4-H), 7.60 (d, *J* = 5.6 Hz, 1H, 2-H), 7.55 (d, *J* = 2.6 Hz, 1H, 2′-H), 7.48 (d, *J* = 5.6 Hz, 1H, 3-H), 7.35 (d, *J* = 8.7 Hz, 1H, 7′-H), 7.21 (d, *J* = 8.6 Hz, 1H, 6-H), 6.78 (dd, *J* = 7.21, 2.2 Hz, 1H, 7-H), 6.67 (dd, *J* = 8.7, 2.3 Hz, 1H, 6′-H), 6.53 (d, *J* = 2.2 Hz, 1H, 9-H), 6.33 (d, *J* = 2.3 Hz, 1H, 4′-H); *m/z* (ESI) 369.47 ($M-H^+$).

*10-(6-Hydroxy-1H-indol-3-yl)-9H-thieno[3,2-b]carbazol-7-ol (**4i**).* Yield 38%, grey powder; mp 193-196 °C; ^1H NMR (DMSO-d_6) δ = 11.07 (d, *J* = 2.3 Hz, 1H, N′-H), 10.93 (s, 1H, NH), 9.32 (s, 1H, OH), 9.00 (s, 1H, OH), 7.74 (s, 1H, 4-H), 7.55 (d, *J* = 5.5 Hz, 1H, 2-H), 7.46 (d, *J* = 5.5 Hz, 1H, 3-H), 7.44 (d, *J* = 2.3 Hz, 1H, 2′-H), 6.90 (d, *J* = 2.2 Hz, 1H, 7′-H), 6.81 (d, *J* = 8.6 Hz, 1H, 9-H), 6.75 (d, *J* = 2.0 Hz, 1H, 6-H), 6.73 (d, *J* = 8.5 Hz, 1H, 4′-H), 6.43 (d, *J* = 8.5 Hz, 1H, 5′-H), 6.22 (dd, *J* = 8.6, 2.0 Hz, 1H, 8-H); *m/z* (ESI) 369.49 ($M-H^+$).

*8-Benzyloxy-10-(5-benzyloxy-1H-indol-3-yl)-9H-thieno[3,2-b]carbazole (**4j**).* Yield 9%, white powder; mp 193-196 °C; ^1H NMR (DMSO-d_6) δ = 11.48 (d, *J* = 2.6 Hz, 1H, N′-H), 11.05 (s, 1H, NH), 7.84 (s, 1H, 4-H), 7.70 (d, *J* = 2.6 Hz, 1H, 2′-H), 7.64 (d, *J* = 5.5 Hz, 1H, 2-H), 7.54 (d, *J* = 8.9 Hz, 1H, 7′-H), 7.51 (d, *J* = 5.5 Hz, 1H, 3-H), 7.31 (d, *J* = 8.6 Hz, 1H, 6-H), 7.26-7.18 (m, 8H, phenylic H), 7.10 (dd, *J* = 6.4, 1.8 Hz, 2H, *o*-phenylic H), 6.98 (dd, *J* = 8.6, 2.7 Hz, 1H, 7-H), 6.97 (dd, *J* = 8.9, 2.6 Hz, 1H, 6′-H), 6.66 (d, *J* = 2.7 Hz, 1H, 9-H), 6.59 (d, *J* = 2.6 Hz, 1H, 4′-H), 4.76 (q, *J* = 14 Hz, 2H, OCH_2), 4.52 (q, *J* = 17 Hz, 2H, OCH_2); *m/z* (ESI) 549.22 ($M-H^+$).

*7-Benzyloxy-10-(6-benzyloxy-1H-indol-3-yl)-9H-thieno[3,2-b]carbazole (**4k**).* Yield 27%, yellow powder; mp 244-247 °C; ^1H NMR (DMSO-d_6) δ = 11.31 (d, *J* = 2.5 Hz, 1H, N′-H), 11.13 (s, 1H, NH), 7.81 (s, 1H, 4-H), 7.59 (d, *J* = 5.5 Hz, 1H, 2-H), 7.56 (d, *J* = 2.5 Hz, 1H, 2′-H), 7.51-7.46 (m, 2H, *o*-phenylic H), 7.49 (d, *J* = 5.5 Hz, 1H, 3-H), 7.47-7.40 (m, 2H, *o*-phenylic H), 7.44-7.25 (m, 6H, *m*-, *p*-phenylic H), 7.13 (d, *J* = 2.3 Hz, 1H, 7′-H), 6.98 (d, *J* = 2.2 Hz, 1H, 6-H), 6.88 (d, *J* = 8.7 Hz, 1H, 9-H), 6.84 (d, *J* = 8.6 Hz, 1H, 4′-H), 6.65 (dd, *J* = 8.6, 2.3 Hz, 1H, 5′-H), 6.46 (dd, *J* = 8.7, 2.2 Hz, 1H, 8-H), 5.15 (s, 2H, OCH_2), 5.12 (s, 2H, OCH_2); *m/z* (ESI) 549.22 ($M-H^+$).
